# Physiological changes of microalga *Dunaliella parva* under the treatment of PEG, CaCl_2_

**DOI:** 10.1371/journal.pone.0295973

**Published:** 2023-12-15

**Authors:** Qiman Zou, Limei Huang, Jinghui Gu, Bingbing Pang, Changhua Shang

**Affiliations:** Key Laboratory of Ecology of Rare and Endangered Species and Environmental Protection (Ministry of Education) & Guangxi Key Laboratory of Landscape Resources Conservation and Sustainable Utilization in Lijiang River Basin, Guangxi Normal University, Guilin, Guangxi, China; Zhengzhou University, CHINA

## Abstract

Carotenoids are antioxidants, which reduce various chronic diseases of human, and have many industrial applications. The halophilic *Dunaliella parva* (*D*. *parva*) is rich in carotenoids. The compounds CaCl_2_ and PEG are the popular metabolic enhancers. To further enhance carotenogenesis, *D*. *parva* was treated with two compounds polyethylene glycol (PEG) and CaCl_2_. Application of CaCl_2_ and PEG enhanced the carotenoids contents and the antioxidant activities of carotenoids compared to control group (no treatment of CaCl_2_ or PEG). The highest carotenoids contents were obtained by treating *D*. *parva* with 40 ppm CaCl_2_ (3.11 mg/g dry weight, DW) and 80 ppm PEG (2.78 mg/g DW) compared with control group (1.96 mg/g DW). When *D*. *parva* was treated with 40 ppm CaCl_2_ and 80 ppm PEG, protein contents reached the highest values (90.28 mg/g DW and 89.57 mg/g DW) compared to that of control group (73.42 mg/g DW). The antioxidant activities of carotenoids samples were determined. Generally, the antioxidant activities of carotenoids from *D*. *parva* treated with PEG and CaCl_2_ were superior to that of control group. The antioxidant activities of carotenoids mainly contained reducing power, hydroxyl radical scavenging activity and superoxide radical scavenging activity. The reducing powers of carotenoids extracts from 20 ppm CaCl_2_ group (2.07%/mg carotenoids) and 120 ppm PEG group (1.59%/mg carotenoids) were significantly higher than that of control group (<1.25%/mg carotenoids). The superoxide radical scavenging activities of carotenoids extracts from 40 ppm CaCl_2_ group (70.33%/mg carotenoids) and 80 ppm PEG group (65.94%/mg carotenoids) were significantly higher than that of control group (<55%/mg carotenoids). This paper laid a foundation for massive accumulation of carotenoids in microalga *D*. *parva*.

## 1. Introduction

Carotenoids are good natural antioxidants that play a key role in the photosynthetic metabolic pathway in the case of light damage caused by excessive light in plant, and are closely related to human health, due to their strong activity of reducing various chronic diseases and enhancing immune regulation [1,2]. The high economic value of carotenoids prompted researchers to increase carotenoid biosynthesis through physical and chemical methods, and genetic engineering techniques. The scientists have paid high attention to microalgae as a source of high-value natural products such as carotenoids [3–5]. Carotenoids accumulation can be affected by various environmental factors. The accumulation of carotenoids was significantly enhanced in microalgae under high temperature, light, and salt stress conditions [6,7]. The blue light and reactive oxygen species could promote the accumulation of carotenoids in *Bifidobacterium tridspora* [8]. The degradation of carotenoid cleavage dioxygenase (CCD) was inhibited by RNA interference to increase carotenoid content [9]. Nitric oxide and salt treatments could improve carotenoid content in crocus [10].

Carotenoid biosynthesis under stress is essential to the improvement of adaptability and resistance. Calcium was widely considered to be one of the most essential nutrients for regulating growth and development, increasing biomass, and enhancing antioxidant activity [11]. Calcium also played an important role in many cellular processes such as signal transduction, and cell membrane stabilization [12]. Calcium could influence growth, development, and lipid accumulation in *Scenedesmus* sp. [13]. However, higher stress of calcium ion resulted in a severe decrease in cell density and carotenoid content, and antioxidant activity in microalgae [14]. UV-B and CaCl_2_ treatments effectively promoted both carotenoids content and antioxidant activities in germinated corn kernels, and CaCl_2_ could further reduce photooxidative damage resulted from UV-B radiation [15]. NaCl and CaCl_2_ could enhance carotenoid accumulation by increasing expression of several carotenogenic genes, and improve antioxidant capacity in germinated yellow maize kernels [16]. Treatment with the nutrient solution including 15 mM CaCl_2_, significantly improved photosynthetic pigment contents and zucchini growth under both normal condition and Ni stress condition [17]. In addition, polyethylene glycol (PEG), a kind of water-soluble polymer, might lead to drought stress in plant cells [18]. Many studies had shown that carotenoid content increased significantly under osmotic stress and salt stress. Carotenoid contents in three carrot cultivars ’Kurodagosun’, ’Benhongjinshi’, and ’Qitouhuang’, were enhanced after 15% PEG-6000 treatment [19]. Pretreating the microalga *Chlorella vulgaris* with PEG could enhance the activities of antioxidant enzymes such as catalase activity, ascorbate peroxidase activity, and superoxide dismutase at all used concentrations, and had a lower rate of chlorophylls loss [20]. The decline of chlorophylls, carotenoids, DPPH radical scavenging capacity, and reducing power was found in leaf discs of tea treated with PEG solutions [21]. Therefore, carotenoid accumulation was thought to be a protective mechanism of algal cells against various stresses.

However, the researches on the effects of CaCl_2_ and PEG focused on plants. The related researches are relatively rare in microalgae. In this study, carotenoids content and its antioxidant activities were investigated under the treatment of metabolic enhancers like CaCl_2_, PEG in *D*. *parva*. This study laid a foundation for understanding the effects of exogenous substances (CaCl_2_, PEG) on carotenoid synthesis in the future.

## 2. Materials and methods

### 2.1. Microalga and growth condition

*D*. *parva* FACHB-815 was purchased from Freshwater Algae Culture Collection at the Institute of Hydrobiology (Wuhan, China). Cells of *D*. *parva* were cultured in Dm medium described by our previous study [6,22]. *D*. *parva* was cultured under light intensity of 34 μmol photons/(m^2^xs) with day-night cycle (14 h light and 10 h dark) at 25°C. The microalgal cells were gently shaken three times each day by hand.

### 2.2. Experimental design

The microalgal mother inoculum was added at 10–20% (v/v) for all experiments. The mother cultures were inoculated into Dm medium with different concentrations of PEG-6000 (20, 40, 80 and 120 ppm), CaCl_2_ (20, 40, 80 and 120 ppm), respectively. The cells were observed for a period of 16 d with three replicates. The biomass was collected to analyze each parameter like pigment content, carbohydrate content, protein content, for mRNA level by fluorescence quantitative PCR, and antioxidant activity. The transgenic microalga was inoculated into Dm medium with the extra chloramphenicol (60 ng/μl), and carotenoids content was monitored every other day.

### 2.3. Growth analysis

The growth curve (OD_600_) was monitored using Microplate Reader (Switzerland, Tecan) every two days until 16 d. To determine the dry weight, algal cells were collected by centrifugation at 4000 ×g for 10 min, then the pellets were dried at 60 ˚C. The unit of dry weight was mg/L. All measurements were repeated three times.

### 2.4. Determination of contents of carotenoid and chlorophyll

For the biochemical analysis, the algal cultures were harvested after 16 d by centrifugation at 4000 ×g for 5 min. After centrifugation, cells were suspended and extracted in 1 ml dimethyl sulfoxide (DMSO) and incubated at 55 ˚C for 30 min. This step was repeated until the adequate extraction. Then pigment production was monitored at 665 nm, 649 nm and 480 nm using Microplate Reader. Pigment content was calculated using the following formula according to the previous study [12]. Pigmentation content expressed as mg/g DW.


$$C_{a}=12.47\times A_{665}‐3.62\times A_{649}$$

$$C_{b}=25.06\times A_{649}‐6.5\times A_{665}$$

$$Cart=(1000\times A_{480}‐1.29\times C_{a}‐53.78\times C_{b})/220$$

C_a_, C_b,_ and Cart represent the concentrations of Chl a, Chl b, and carotenoids in DMSO extract (mg/l), respectively, and A_665_, A_649,_ and A_480_ are the absorbance of carotenoid extract at the corresponding wavelength. The final carotenoid content (mg/g) DW is calculated by the following formula. “Chl a”, “Chl b” or “Carotenoids” = [“C_a_”, “C_b_” or “Cart” (mg/L)×volume (L)]/dry weight of the sample (g).

### 2.5. Carbohydrate and protein contents

Total sugar content of alga was determined by anthrone colorimetry [23]. Protein content was determined by Coomassie Brilliant Blue G-250 method using bovine serum albumin as standard.

### 2.6. Fluorescence quantitative PCR (FQ-PCR) analysis

To clarify the expression of key enzyme genes, several genes including *DpAP2*, *PSY*, *PSD* and *GGPS* were selected for quantitative analysis using several pairs of primers (S1 Table) according to the manufacturer’s instruction. Briefly, total RNA was extracted using MiniBEST Plant RNA Extraction Kit (Takara) and used to synthesize first-strand cDNA for FQ-PCR. Reaction mixture was 20 μl, which included 10 μL of 2×SuperReal Premix, 0.6 μl of sense primer (10 μM), 0.6 μl of antisense primer (10 μM), 1 μl of cDNA. PCR procedure was as follows: 95 ˚C for 15 min, (95 ˚C for 10 s, 60 ˚C for 30 s, 40 cycles).

### 2.7. Antioxidant activity of extracted carotenoids

Carotenoids were extracted from *D*. *parva* by ethanol and evaporated at 55 ˚C until the concentration reached a certain value, then used for measuring antioxidant activity. Three main antioxidant activity indices closely related to carotenoids content were determined, which included reducing power, hydroxyl radical scavenging activity, and superoxide radical scavenging activity.

The method of Ismaiel et al. (2021) [24] was used to analyze reducing power of carotenoids extract. Hydroxyl radical scavenging activity of carotenoid extract was determined based the method of Smirnoff and Cumbes (1989). Superoxide radical scavenging activity of carotenoid extract was determined in riboflavin-light-NBT system based the method of Beauchamp and Fridovici (1971) [25].

### 2.8. Statistical analysis

The measured data were expressed as mean±standard error (SE) based on three independent experiments. Statistical analyses were performed using SPSS 10.0 software. The graphics were constructed based on measured data using Origin 2022 software. Analysis of variance (ANOVA) with Duncan’s multiple range tests was used to compare significance level [12].

## 3. Results

### 3.1. Growth of *D*. *parva*

**Fig 1:**
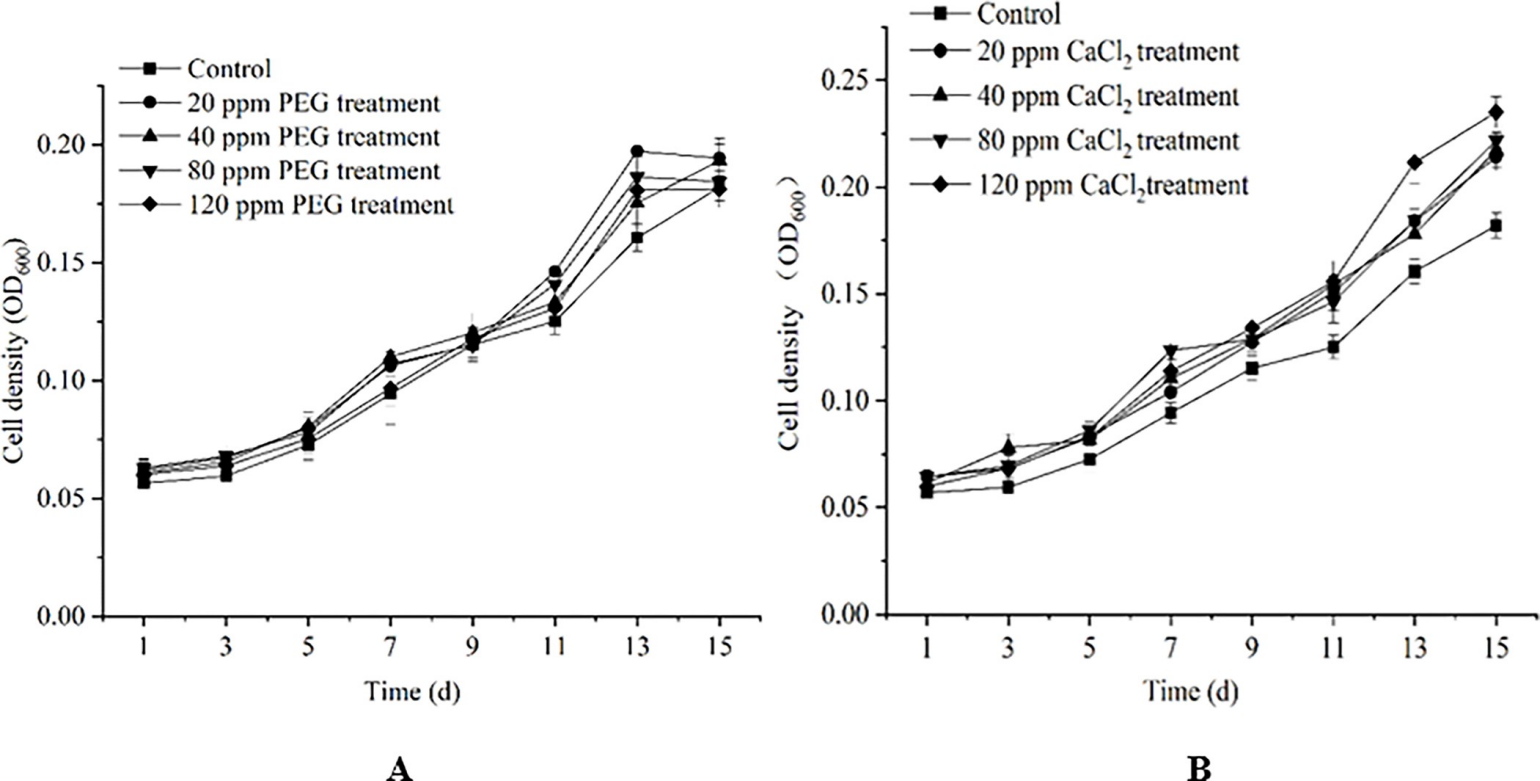
Cell density of *D*. *parva* under different concentrations of PEG-6000 (A), CaCl_2_ (B). The values represent mean±SE of three replicates.

The density of algal cells treated with different concentrations of exogenous substances (PEG, CaCl_2_) was determined. There was a significant increase in cell density for cultures treated with different concentrations of PEG compared with control group from 11d to 13d (Fig 1A). Cell density (0.1973) of group treated with PEG (20 ppm) was significantly higher than that of control group (0.1615) at 13d, which increased by 22.17%. The growth of groups treated with different concentrations of CaCl_2_ was better than that of control group (Fig 1B). The effects of CaCl_2_ and PEG-6000 on cell density were similar. The results showed that the highest cell density (0.2351) was observed in cells treated with CaCl_2_ (120 ppm) at 15 d, which increased by 29.25% compared with cell density of control group (0.1819).

### 3.2. Carotenoids content

**Fig 2:**
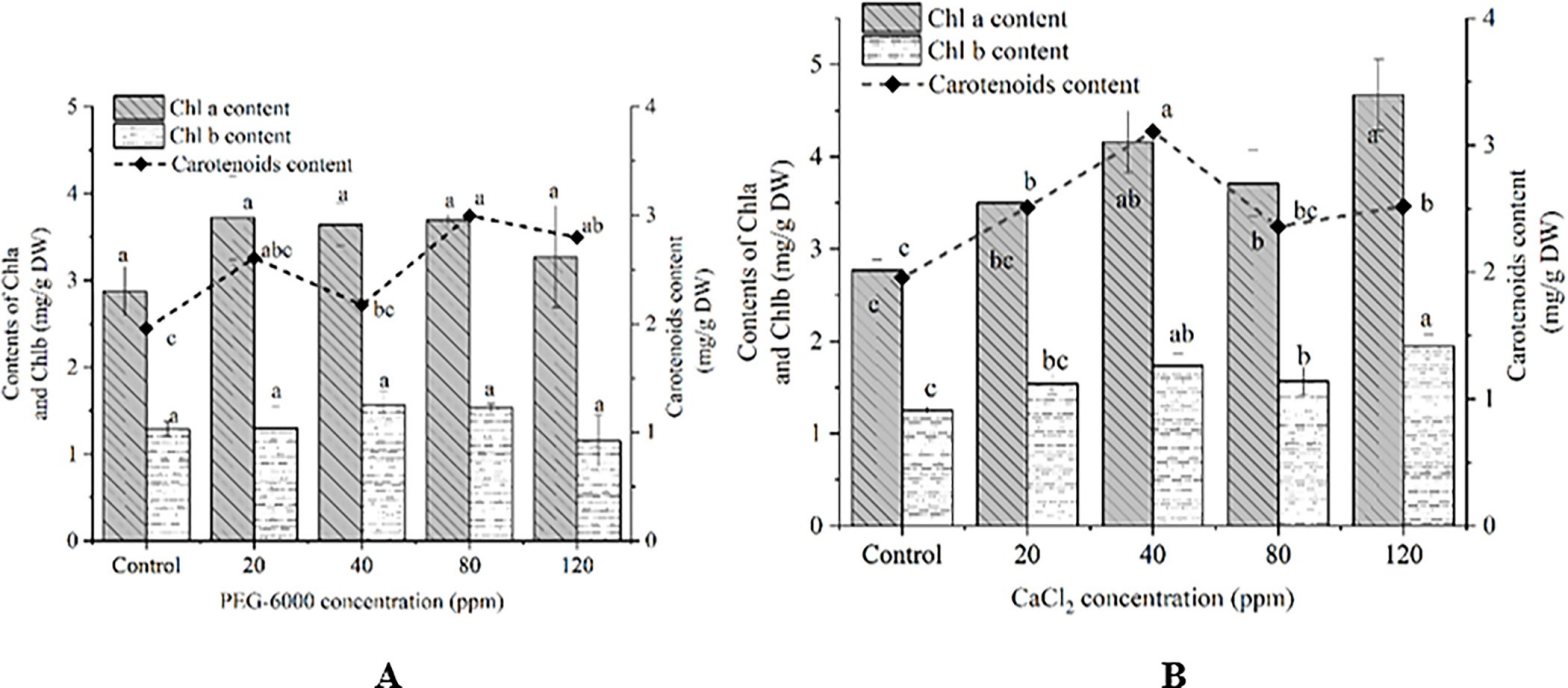
Chlorophyll a, b (Chl a, Chl b) and carotenoids contents of *D*. *parva* cells under treatment of various concentrations of PEG-6000 (A), CaCl_2_ (B). The bars represent mean±SE of three replicates. The different letters represent significant differences (*P*<0.05).

As carotenoids, Chl a and Chl b synthesis will influence other metabolic processes, their contents were evaluated at 16 d (Fig 2). After 80 ppm PEG treatment, the highest carotenoid content reached 2.78 mg/g DW, followed by 120ppm PEG treatment (2.69mg/g DW), which increased by 41.83% and 37.24% compared with control group (1.96 mg/g DW) at 16d. In addition, PEG (20ppm, 40ppm) also stimulated carotenoid biosynthesis (2.60, 2.17 mg/g DW). However, no significant difference in Chl a and Chl b contents was found in PEG-treated cultures (Fig 2A).

Carotenoid contents of CaCl_2_-treated cell cultures were significantly higher compared with control group (Fig 2B). The contents of Chl a & Chl b were significantly enhanced by the treatment of CaCl_2_ concentrations (40 ppm, 120 ppm), followed by CaCl_2_ (80 ppm). Carotenoid content of 40 ppm CaCl_2_ treated group reached 3.11 mg/g DW, which was significantly higher than that of control group (1.96 mg/g DW), increasing by 58.67%.

### 3.3. Carbohydrate and protein contents

**Fig 3:**
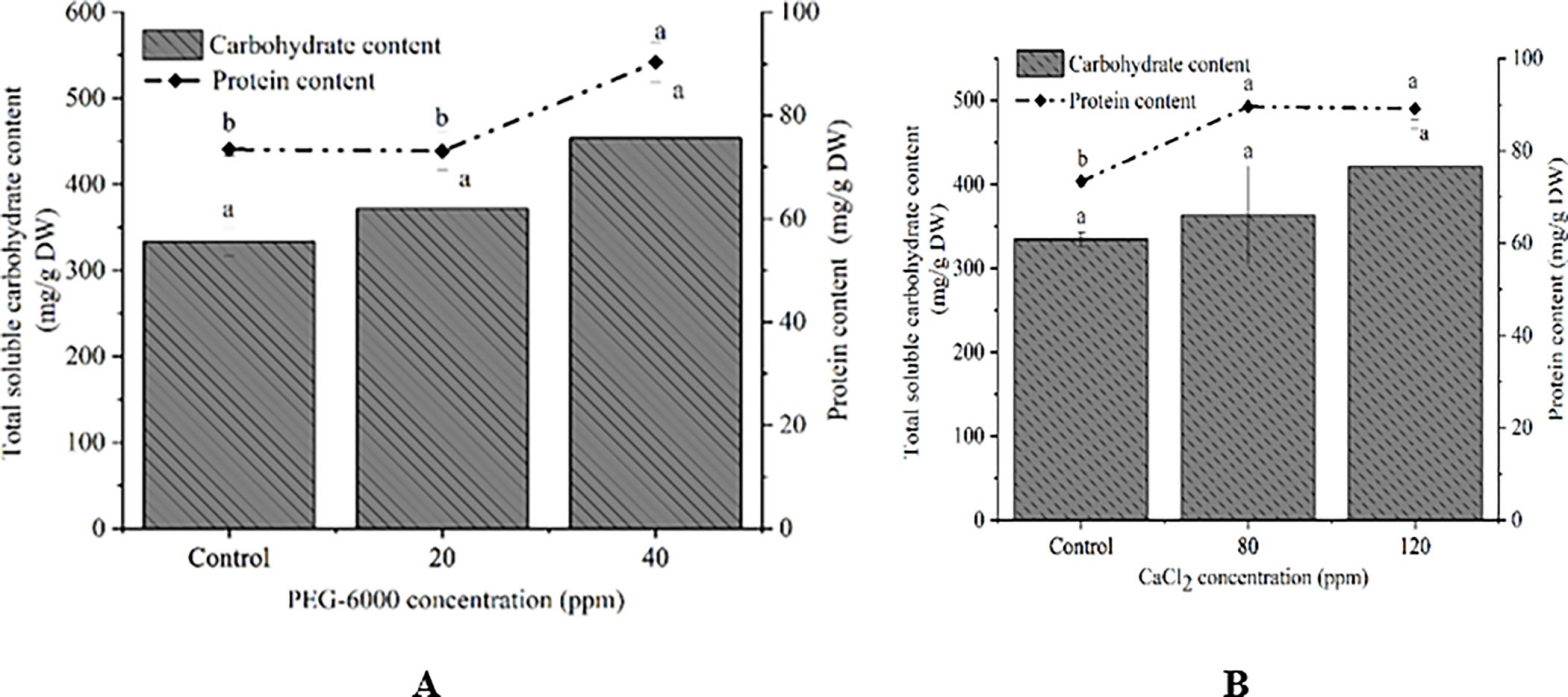
Total soluble carbohydrate contents and protein content of *D*. *parva* cells with the treatment of PEG-6000 (A), CaCl_2_ (B). The bars represent mean±SE of three replicates. The different letters represent significant differences (*P*<0.05).

The influence of CaCl_2_ and PEG on the contents of carbohydrate and protein were shown in Fig 3. The results showed that there was a significant increase in protein content, which was similar to the results of carotenoid content. The protein content (89.57, 89.15 mg/g DW) was significantly enhanced due to the treatment of PEG (80 ppm, 120 ppm) compared with protein content of control group (Fig 3A). The largest protein content of cells treated with 40ppm CaCl_2_ could reach 90.28 mg/g DW, which increased by 16.86 mg/g DW compared with control group (73.42 mg/g DW) (Fig 3B). Carbohydrate contents of groups treated with 40 ppm CaCl_2_ (453.61 mg/g DW), 120 ppm PEG (421.32 mg/g DW), 20 ppm CaCl_2_ (371.88 mg/g DW) and 80 ppm PEG (363.14 mg/g DW) were higher compared with control groups (CaCl_2_ control, 333.39 mg/g DW, PEG control, 334.46 mg/g DW).

### 3.4. Fluorescent quantitative PCR analysis

**Fig 4:**
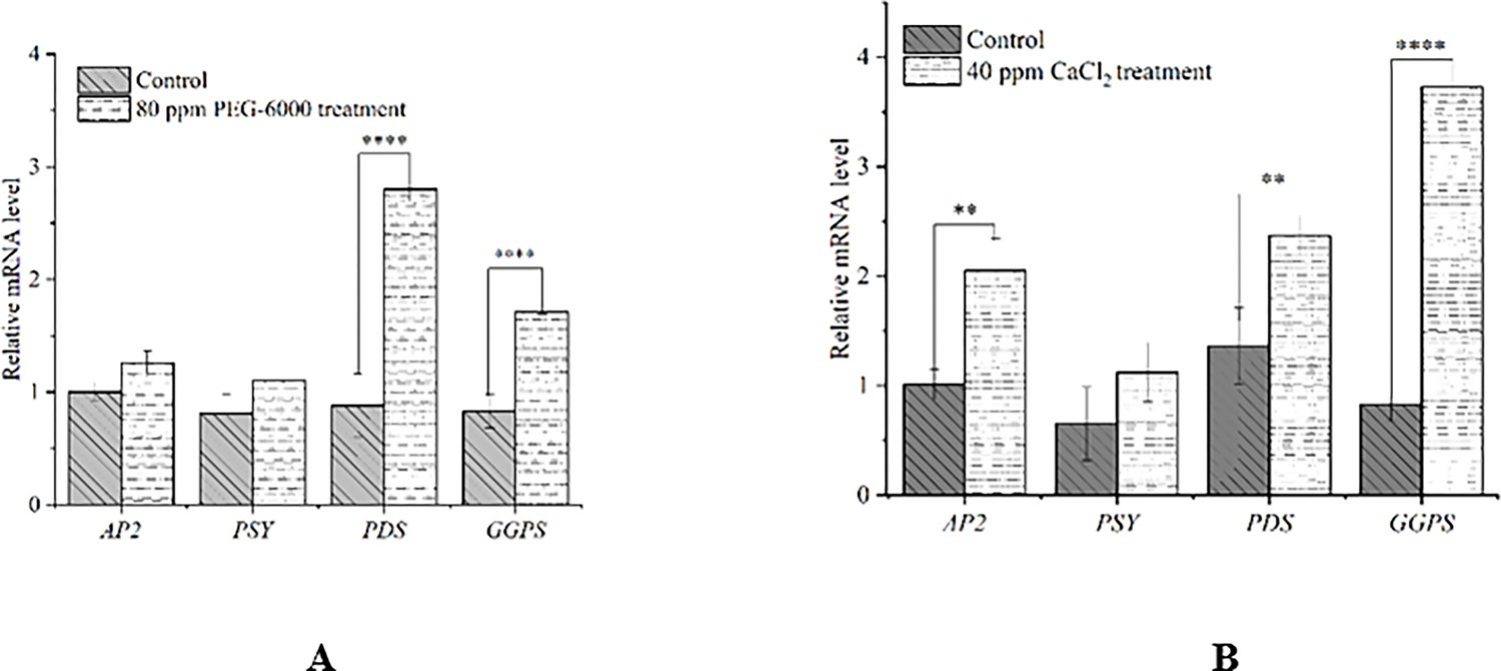
Relative mRNA levels of *D*. *parva* cells under the treatment of PEG-6000 (A), CaCl_2_ (B). The bars represent mean±SE of three replicates. The symbols “*”, “**”, “***” and “****” represent *P*<0.05, *P*<0.01, *P*<0.001 and *P*<0.0001, respectively.

Previous studies had found that four key genes *AP2*, *PSY*, *PDS* and *GGPS* involved in the regulation of carotenoid synthesis pathway in plants, however, little was known about the function of four genes in *D*. *parva*. The *GGPS*, *PSY*, and *PDS* genes encode geranylgeranyl diphosphate synthase, phytoene synthase, and phytoene desaturase, respectively. The relative expressions of *DpAP2*, *PDS* and *GGPS* with CaCl_2_ stress treatment significantly increased (Fig 4B), and the relative expressions of *PDS* and *GGPS* with PEG stress treatment significantly increased (Fig 4A).

### 3.5. Reducing power

**Fig 5:**
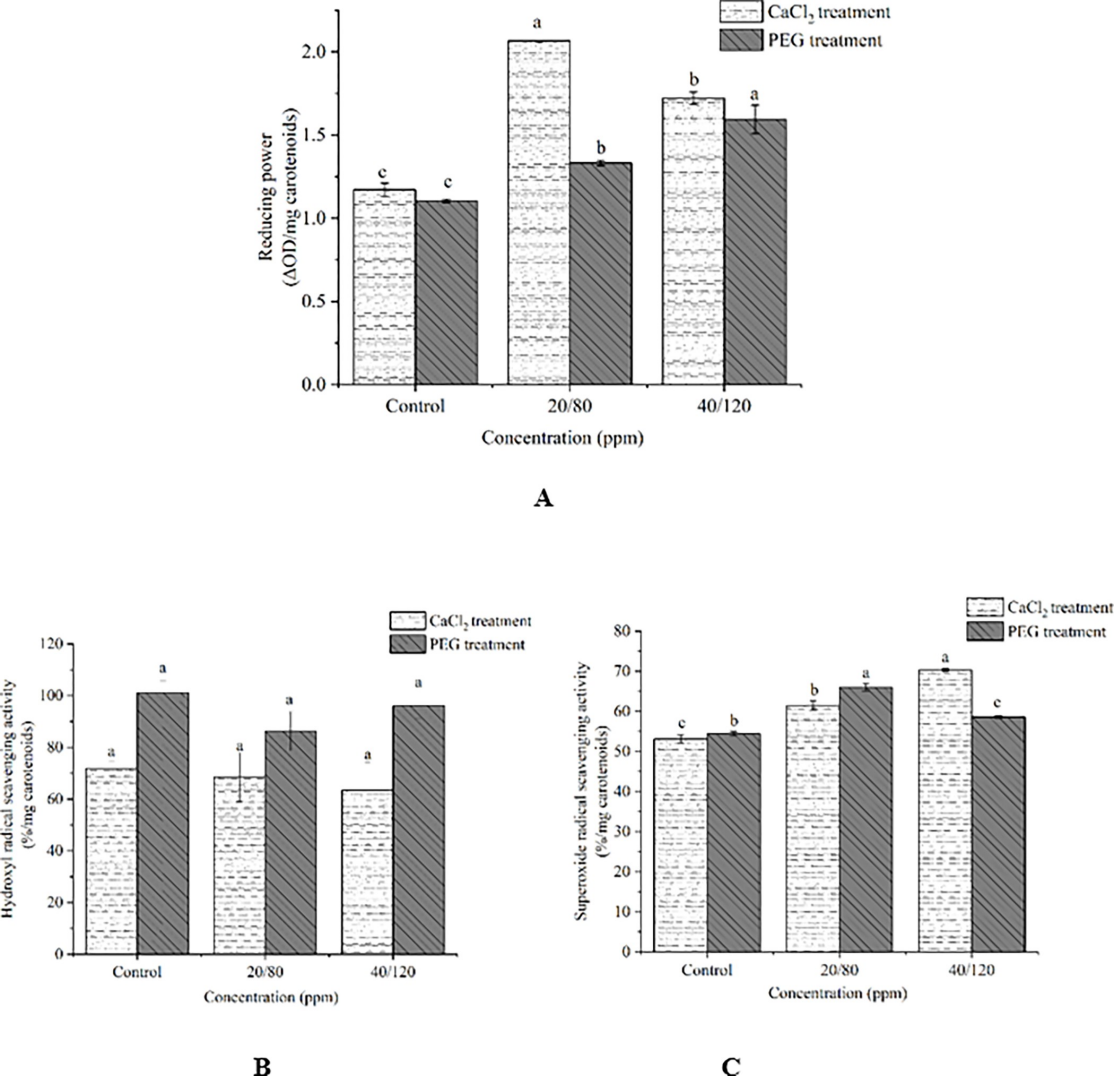
Antioxidant activities of carotenoids extracts (reducing power, A; hydroxyl radical scavenging activity, B; superoxide radical scavenging activity, C) under the treatment of PEG-6000 (80ppm,120ppm) and CaCl_2_ (20ppm, 40ppm). The bars represent mean±SE of three replicates.

The reducing power of carotenoids extract was determined under the treatment of exogenous substances like 40/20 ppm CaCl_2_ and 80/120 ppm PEG (Fig 5A). The reducing powers of 20 ppm CaCl_2_ group (2.07%/mg carotenoids) and 40 ppm CaCl_2_ group (1.72%/mg carotenoids) were significantly higher than that of control group (1.17%/mg carotenoids). The reducing powers of carotenoids extract (1.33%, 1.59%/mg carotenoids) were enhanced due to the effects of PEG (80 ppm, 120 ppm). The reducing powers of carotenoids extract under treatment of CaCl_2_ and PEG increased by 0.90% and 0.49%/mg carotenoids compared with control group, respectively.

### 3.6. Hydroxyl radical scavenging activity

The results showed that there was no distinct difference in hydroxyl radical scavenging activity of carotenoids extract under the treatment of diverse concentrations of CaCl_2_ and PEG (Fig 5B). The largest hydroxyl radical scavenging activity was up to 101.16%/mg carotenoids without any exogenous substance, which only increased by 14.86%, 5.08%/mg carotenoids compared with 80 ppm PEG group (86. 30%/mg carotenoids) and 120 ppm PEG group (96.08%/mg carotenoids), respectively. The difference between 20 ppm CaCl_2_ group (68.50%/mg carotenoids)/40 ppm CaCl_2_ group (63.44%/mg carotenoids) and control group (71.76%/mg carotenoids) were 3.26% and 8.32%/mg carotenoids, respectively.

### 3.7. Superoxide radical scavenging activity

The results showed a significant increase in superoxide radical scavenging activity of carotenoids extract under the treatment of CaCl_2_ and PEG, which were similar to the results of reducing power (Fig 5C). The largest superoxide radical scavenging activity of carotenoids extract reached 70.33%/mg carotenoids under the treatment of 40 ppm CaCl_2_, which enhanced 17.24% compared with control group (53.09%/mg carotenoids). Superoxide radical scavenging activity of 80 ppm PEG group (65.94%/mg carotenoids) increased by 11.50% compared with that of control group (54.44%/mg carotenoids).

## 4. Discussion

PEG and CaCl_2_ are known to cause osmotic stress and nutrient stress which promote the growth and carotenoid biosynthesis in plants. Polyethylene glycol (PEG) is a water-soluble polymer that can simulate drought stress induction in plants. Drought stress can lead to the production and accumulation of free radical in algal cells, which leads to the peroxidation of membrane lipids and the decline of membrane stability. However, carotenoids have the activity of scavenging hydrogen peroxide, hydroxyl radical, superoxide radical and singlet oxygen, which can delay lipid peroxidation and ultimately protect cells from oxidative damage [12,26]. In the previous study, a significant increase in chlorophylls a/b and carotenoids contents under the treatment of 30 ppm PEG and 60 ppm CaCl_2_ was reported. In addition, carotenoids of *D*. *parva* with the treatment of PEG and CaCl_2_ had remarkably higher antioxidant activities than that of control group [12]. In contrast, the contents of chlorophylls (a and b) and carotenoids significantly decreased under the treatment of various PEG-6000 concentrations (w/v, 5%, 10%, 20%, 30%) in maize leaves. The reduction of fresh was reported under the treatment of different PEG concentrations (5%, 10% and 15%) [27]. The reason of this phenomenon may be associated with a decrease in cellular water content (water stress) caused by PEG treatment [28]. Calcium (Ca^2+^) is an essential nutrient for stabilizing the structure of cell wall, regulating signal transduction and ion transport, which also plays a key role in cellular functions by binding proteins and activating the activity of target proteins [29]. The production of carotenoid, indole-3-acetic acid, DPPH, chlorophyll, and relative water content were improved under Ca^2+^ stress in cultivar of canola (Sarigol) [30]. Similarly, Ca^2+^ plays an essential role in the antioxidant activities such as the activities of catalase and SOD in cultivar of canola (RGS003) under drought stress after 24 h [27]. In our experiment, different concentrations of PEG-6000 (20, 40, 80, 120ppm) and CaCl_2_ (20, 40, 80, 120ppm) were used to treat algal cells. It was found that carotenoids content reached the highest values of 3.11mg/g DW and 2.78mg/g DW under the treatment of 40 ppm CaCl_2_ and 80 ppm PEG, which increased by 58.67% and 41.84% compared with control group (1.96mg/g DW). Carotenoids are synthesized from several steps such as desaturation, cyclization, hydroxylation and epoxidation within the plastids [22]. Key enzymes of carotenoids synthesis pathway include *DXS*, *DXR*, *GGPS*, *PSY* and *PDS*. The relative expression levels of *PDS* and *GGPS* increased significantly under PEG stress, which promoted the accumulation of carotenoids. The relative expressions of *DpAP2*, *PDS* and *GGPS* increased significantly under CaCl_2_ treatment, which promoted the accumulation of carotenoids.

Carotenoids are natural antioxidants, which play a key role in the photosynthetic metabolic pathway in the case of light damage caused by excessive light. To reflect the power of carotenoids, we reported three main antioxidant activities closely related to carotenoids content including reducing power, hydroxyl radical scavenging activity and superoxide radical scavenging activity. The ability of carotenoids to reduce Fe^3+^ to Fe^2+^ may indirectly capitalize on reducing power of antioxidant activity [12]. This study showed that reducing power was significantly higher than control group under the treatment of 40/20 ppm CaCl_2_ and 80/120 ppm PEG. The cause of this phenomenon may be associated with CaCl_2_ and PEG which further reduce Fe^3+^ to Fe^2+^. SOD can catalyze the transformation of superoxide free radical into molecular oxygen and hydrogen peroxide under salt and PEG stresses [31]. Carotenoids were known to have the activity of SOD, which further detoxified cells and protect them from the damage of biochemical substances. In this paper, the antioxidant activity like superoxide radical scavenging activity was similar to reducing power, which was significantly higher than control group with the treatment of metabolic enhancers like CaCl_2_ and PEG. Therefore, it can be inferred that superoxide radical scavenging activity of carotenoids extract increased after CaCl_2_ and PEG treatment.

## 5. Conclusion

Application of CaCl_2_ and PEG enhanced carotenoids contents, protein contents, and the antioxidant activities of carotenoids compared to control group (no treatment of CaCl_2_ or PEG). Generally, the antioxidant activities of carotenoids from *D*. *parva* treated with PEG and CaCl_2_ were superior to that of control group. The contents of carotenoids and protein, and antioxidant activities were markedly higher than that in control group under the treatment of exogenous substances (20 ppm/40 ppm CaCl_2_, 80 ppm/120 ppm PEG). Carotenoid biosynthesis was enhanced by exogenous substances such as CaCl_2_, PEG, which laid a foundation for improving the economic value of *D*. *parva* in the pharmaceutical industry.

## Supporting information

S1 Table The sequence of the primers. (DOCX)
