# Supplementary material for: Physiological changes of microalga Dunaliella parva under the treatment of PEG, CaCl2
Source: PLoS One. 2023 Dec 15;18(12):e0295973. doi: 10.1371/journal.pone.0295973 (PMC10723680; doi:10.1371/journal.pone.0295973)
Supplement: S1 Table — (DOCX) [file pone.0295973.s001.docx]

**Supporting information**

**S1 Table. The sequence of the primers.**

| **Primer** | **Sequence (5′-3′)** |
| --- | --- |
| AP2(Real-R) | GGCAGCCAGGTCATAACTCA |
| AP2(Real-F) | CGCACCAACAGATGGGAGT |
| Pds(Real-R) | TTGTACTTGCGGATCTTGG |
| Pds(Real-F) | GCTGGAGCTGGTGTTCG |
| Psy(Real-R) | CATCAAGGATCTGGCGGTA |
| Psy(Real-F) | GGCAGGCATCCACAAGC |
| Ggps(Real-R) | CCCCAGCTCACAGATCACA |
| Ggps(Real-F) | GAGGGAACCCAACAAACCA |
